# Adhesion Molecule Expression and Function of Primary Endothelial Cells in Benign and Malignant Tissues Correlates with Proliferation

**DOI:** 10.1371/journal.pone.0091808

**Published:** 2014-03-14

**Authors:** Wolfgang Sievert, Soile Tapio, Stephanie Breuninger, Udo Gaipl, Nicolaus Andratschke, Klaus-Rüdiger Trott, Gabriele Multhoff

**Affiliations:** 1 Department of Radiation Oncology, Klinikum rechts der Isar, Technische Universität München, Munich, Germany; 2 Clinical Cooperation Group (CCG) “Innate Immunity in Tumor Biology”, Helmholtz Zentrum München (HMGU), German Research Center for Environmental Health Munich, Neuherberg, Germany; 3 Department of Radiation Biology, Helmholtz Zentrum München (HMGU), German Research Center for Environmental Health Munich, Neuherberg, Germany; 4 Department of Radiation Oncology, University of Erlangen-Nürnberg, Erlangen, Germany; 5 Department of Oncology, Imperial University College London, London, United Kingdom; Nottingham Trent University, United Kingdom

## Abstract

**Background:**

Comparative analysis of the cellular biology of the microvasculature in different tissues requires the availability of viable primary endothelial cells (ECs). This study describes a novel method to isolate primary ECs from healthy organs, repair blastemas and tumors as examples of non-proliferating and proliferating benign and malignant tissues and their functional characterization.

**Methodology/Principal Findings:**

Single cell suspensions from hearts, lungs, repair blastemas and tumors were incubated consecutively with an anti-CD31 antibody and magnetic micro-beads, coupled to a derivative of biotin and streptavidin, respectively. Following magnetic bead separation, CD31-positive ECs were released by biotin-streptavidin competition. In the absence of micro-beads, ECs became adherent to plastic surfaces. ECs from proliferating repair blastemas and tumors were larger and exhibited higher expression densities of CD31, CD105 and CD102 compared to those from non-proliferating normal tissues such as heart and lung. The expression density of CD34 was particularly high in tumor-derived ECs, and that of CD54 and CD144 in ECs of repair blastemas. Functionally, ECs of non-proliferating and proliferating tissues differed in their capacity to form tubes in matrigel and to align under flow conditions.

**Conclusions/Significance:**

This method provides a powerful tool to generate high yields of viable, primary ECs of different origins. The results suggest that an altered expression of adhesion molecules on ECs in proliferating tissues contribute to loss of EC function that might cause a chaotic tumor vasculature.

## Introduction

The diffusion limit of oxygen from the capillary to non-vascular tissue in the body ranges from 100 to 200 μm. Therefore, a dense network of blood vessels is necessary to provide an adequate supply of oxygen and nutrients [1], [2]. In capillaries, the endothelial monolayer is the only cell barrier between blood and intercellular space, stroma and parenchymal cells. Microvascular ECs also fulfill important functions in wound healing and blood flow regulation e.g. by preventing thrombosis. Although in adult organs, the turnover rate of ECs is generally slow [3], in wound healing, in the female reproductive cycle and during pregnancy the proliferation of ECs is very high [2], [4]. In tumors, the proliferative capacity of ECs may be a limiting factor for the growth of tumors [5]. It is also known that the microvascular architecture of tumors differs from that of normal tissues. Tumor vessels develop fewer branches, are often tortuous and have variable diameters and a higher permeability [6].

In contrast to the primary microvasculature, endothelial cell lines proliferate rapidly *in vitro*. Therefore, endothelial cell lines might resemble certain aspects of the tumor microvasculature, but may not be suitable for studies of normal ECs in adult organs and tissues.

To investigate functional properties of primary ECs in non-proliferative normal tissues such as heart or lung or in actively growing benign and malignant tissues such as healing wounds and tumors, an extraction of viable primary ECs from the respective tissue is necessary. This is particularly important in the study of key pathogenic mechanisms of late functional damage to the microvasculature in normal and tumor tissues following ionizing irradiation. On the one hand, low radiation doses of a few Gy can lead to cardiovascular injury, which has been suggested to be related to long-term functional changes in slowly proliferating ECs [7], [8], rather than to a direct killing of ECs, a proliferative failure, or a loss in clonogenicity, all of which are commonly associated with radiation-induced DNA damage. On the other hand, impairment of the proliferative capacity of ECs in the tumor and its microenvironment is a well-established mechanism that results in a retarded re-growth of irradiated tumors [9].

In order to identify phenotypic and functional characteristics of ECs derived from stable, non-proliferating (heart, lung) and proliferating benign (repair blastema) and malignant tissues (tumors) of mice of different ages, a novel method was established that allows the isolation of viable, primary microvascular ECs.

## Methods

### Animals

Female/male BALB/c and C57Bl/6 mice (Charles River) within an age range of 4 to 100 weeks were used for the experiments. Animals were housed in single, ventilated cages under pathogen-free conditions. All experiments were approved by the “Regierung von Oberbayern” and were performed in accordance with institutional guidelines of the Klinikum rechts der Isar, Technische Universität München.

### Established Cell Lines

CT26 mouse colon adenocarcinoma cells (CT26.WT; ATCC CRL-2638) were cultured in RPMI 1640 medium containing 5% v/v heat-inactivated fetal calf serum, 2 mM L-glutamine, antibiotics (100 IU/ml penicillin and 100 μg/ml streptomycin), 50 μM β-mercaptoethanol and non-essential amino acids (1×). B16-F0 mouse melanoma cells (B16-F0; ATCC CRL-6322) and a murine immortalized heart endothelial cell line H5V [10] were cultured in DMEM medium (Life Technologies, Carlsbad, CA, USA) containing 10% v/v heat-inactivated fetal calf serum (PAA Laboratories GmbH, Pasching, Austria) and antibiotics (100 IU/ml penicillin and 100 μg/ml streptomycin, Life Technologies). Cells were passaged twice weekly to maintain them under exponential growth conditions. Cells were regularly screened and confirmed to be free from mycoplasma contaminations using an enzyme immunoassay (Roche Diagnostics, Penzberg, Germany).

### Tumor Implantation and Repair Blastema Induction

In order to establish tumors in vivo, 1×10^5^ viable CT26 or B16-F0 cells were injected s.c. into the backs of anesthetized 10 week-old BALB/c or C57Bl/6 mice. Tumor growth was determined using sonographic measurements (GE Healthare). Mice were sacrificed when the tumor had reached a volume ranging between 0.3 and 0.4 cm^3^. In order to induce a repair blastema, 0.5 ml sterile air and 0.5 ml sterile incomplete Freund’s adjuvant/PBS-emulsion (1∶1) were injected s.c. into the backs of anesthetized 10 week-old C57Bl/6 mice. Three weeks after injection the repair blastema was dissected.

### Isolation of Microvascular ECs from Heart, Lung, Tumor and Repair Blastema

**Figure 1:**
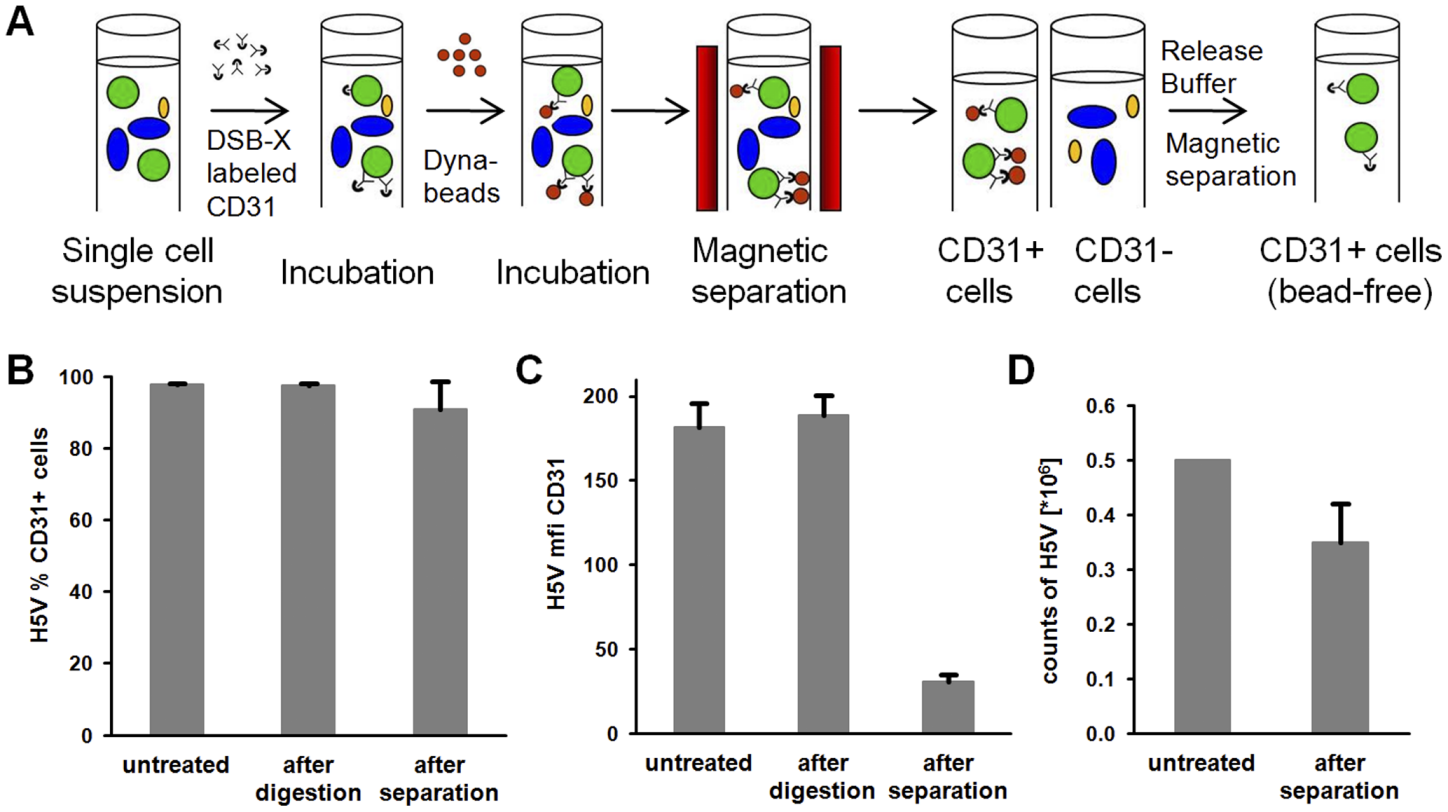
Schematic representation of the isolation procedure of primary, bead-free endothelial cells (ECs). (**A**) First, DSB-X biotin-labeled rat anti-mouse CD31-antibody binds to CD31-positive ECs. Subsequently, streptavidin-labeled Dynabeads bind to DSB-X biotin. After magnetic separation, the Dynabeads are removed by a biotin-streptavidin competition. (**B**) Separation of CD31-positive H5V cells mixed with CD31-negative CT26 tumor cells at a ratio of 20∶1. The proportion (%) of CD31-positive H5V cells and the mean fluorescence intensity (mfi) remained unchanged after a simulated digestion step. (**C**) After separation, the mean fluorescence intensity (mfi) remained stable after digestion (second bar), but dropped to 17% after bead separation (third bar). (**D**) The recovery rate of H5V cells after separation was greater 70%.

For each isolation procedure, one to four mice were sacrificed by craniocervical dislocation. Heart, lung, repair blastema and tumors of the mice were collected under aseptic conditions. To reduce contaminations with macrovascular ECs, the left and right atria were surgically removed from the heart. The tissues were rinsed intensely in ice-cold PBS (Gibco). After mechanical mincing with a sterile scalpel blade, tissue fragments with a size of 1 mm^3^ were transferred into 10 ml of pre-warmed (37°C) digestion solution consisting of collagenase A (0.5 units/ml; Roche Penzberg, Germany) diluted in Hanks’ Balanced Salt Solution (HBSS, Gibco)/10% FCS (PAA). After an incubation period of 45 minutes at 37°C under gentle rotation (2 rpm), the suspension was dispersed by forcing it through a sterile 18 G injection needle 10 times. The single cell suspension was filtered through a 70 μm cell strainer (BD Bioscience) and washed twice in 50 ml HBSS/10% FCS solution with centrifugation at 400×g for 10 min. Following re-suspension of the final cell pellet in 600 μl ice-cold isolation buffer (Invitrogen), DSB-X biotin-labeled (Molecular Probes) rat anti-mouse CD31-antibody (25 μl; 0.5 mg/ml; BD Biosciences, Heidelberg, Germany) was added and incubated for 10 minutes at 4°C under gentle rotation (3 rpm). After a washing step in ice-cold isolation buffer (2 ml), the cell pellet was resuspended in 1 ml cold isolation buffer. FlowComp Dynabeads coated with streptavidin (75 μl; Invitrogen) were added and incubated for a further 15 minutes at 4°C under gentle rotation (3 rpm). The tube was then placed in the magnetic separator (Invitrogen). After 2 minutes, the bead-free, unbound CD31-negative cells were removed from the cell suspension by pipetting. CD31-positive cells immobilized in the DSB-X-streptavidin-bead-complex, remained on the wall of the tube due to the magnetic field (magnet, Invitrogen). After at least 5 washing steps with isolation buffer (1 ml), cells attached to beads were resuspended in 1 ml FlowComp release buffer (Invitrogen) and incubated for 2 minutes at 21°C. By pipetting the cell suspension 10 times, the cells detached from the beads by biotin-streptavidin competition. Then the tube was placed in the magnet separator. After 1 minute, bead-free CD31-positive cells could be isolated, whereas the beads remained on the wall of the tube (Figure 1A).

### Culture of Primary ECs

Freshly isolated primary microvascular CD31-positive ECs were counted and 1×10^6^ cells were seeded in gelatin (2%; Merck) coated T12.5 culture flasks. Endothelial Cell Growth Medium (EGM2, PromoCell) supplemented with 10% FCS, streptomycin (100 μg/ml) and penicillin (100 U/ml) was exchanged every third day and cells were passaged when 90% confluence was reached. For further functional assays, primary ECs in the first or second passage were used.

### Phenotypic Characterization of Primary ECs

Freshly isolated, primary microvascular ECs were phenotypically characterized by flow cytometry on a FACSCalibur instrument (BD, Heidelberg, Germany) using the following fluorescein (FITC), phycoerythrin (PE) or allophycocyanin (APC) conjugated antibodies: CD31 (PECAM-1, BD Bioscience, clone MEC 13.3), CD34 (mucosialin, eBioscience, clone RAM34), CD45 (leukocyte common antigen, BD Bioscience, clone 30-F11), CD54 (ICAM-1, BD Bioscience, clone 3E2), CD61 (integrin β3, BD Bioscience, clone 2C9.G2), CD102 (ICAM-2, BD Bioscience, clone 3C4), CD105 (endoglin, eBioscience, clone MJ7/18) and CD144 (VE-cadherin, BD Bioscience, clone 11D4.1). Appropriately labeled isotype-matched immunoglobulins were used as negative controls. Briefly, 0.1×10^6^ viable cells were incubated with the indicated antibodies for 30 min at 4°C in the dark. Following a washing step in PBS/FCS (10%) cells were analyzed on a FACSCalibur instrument. Dead cells were excluded from the analysis by a propidium iodide co-staining and gating strategy.

### Immunohistochemistry and Immunofluorescence

Formalin-fixed tissue was cut and stained with eosin (eosin y-solution 0.5% aqueos, Merck) and hematoxylin (Mayer’s haematoxylin, Dako). For the CD31 staining primary ECs were seeded into 8-well chamber slides. After adherence, cells were incubated with rat-anti-mouse CD31 (clone MEC 13.3, 1∶100 in primary antibody diluent) for 1 h at 23°C and goat-anti-rat Alexa Fluor 594-labeled secondary antibody (1∶200 in PBS) for 1 h at 37°C. Nuclei were co-stained with Fluorescent Mounting Medium (Dako). For the isolectin staining, cells were blocked with PBS/BSA (0.2%, Sigma) and incubated with isolectin-FITC-labeled antibody (1∶50 in PBS/BSA 0.2%, Vector FL-1201) for 30 minutes at 4°C. Nuclei were co-stained with Vectashield (Vector Labs). In order to detect the distribution of capillaries and the percentage of necrosis/apoptosis, representative microscopy images from the heart and tumor were recorded with Axioskop 2 plus Axio Cam MRc5 (Zeiss).

### Flow System Cell Alignment Assay

For the analysis of heart and tumor ECs with permanent shear stress, ECs were used directly after their isolation. 400,000 cells/channel were seeded into μ-slideVI^0.4^ (IBIDI). After incubation for 2 days at 37°C under static conditions cells were exposed to low flow conditions at 2 ml/min (3.5 dyn/cm^2^). A gas mixer, temperature controller, heating system and pump system (IBIDI) guaranteed constant conditions. After 1 day the flow was increased to 4 ml/min (7 dyn/cm^2^) for additional 4 days and representative microscopy pictures of ECs were taken using an Axiovert 40C microscope (Zeiss).

### Migration and Tube Formation Assays

10,000 cells were seeded into μ-dishes 35 mm, low with Culture-Insert (IBIDI) in a volume of 70 μl EGM2 medium supplemented with 10% FCS, Streptomycin (100 μg/ml) and Penicillin (100 U/ml). Cells were allowed to become adherent at which time the migration of heart and tumor ECs was captured on a video and analyzed. For quantitative evaluation of cell tracking, films were analyzed with WimTaxis (Wimasis, Munich, Germany). For tube formation, each well of a μ-slide Angiogenesis (IBIDI) was filled with 10 μl chilled matrigel (BD). After incubation for 30 min at 37°C, H5V cells (10,000 cells/well), primary heart ECs (80,000 cells/well) or tumor ECs (20,000 cells/well) in passage 1 were added in 50 μl medium. After 6 h, images were recorded with an Axiovert 40C microscope (Zeiss). For quantitative evaluation, representative pictures were analyzed with WimTube (Wimasis, Munich, Germany). The total number of branching points and loops per image were determined.

### Statistical Analysis

Comparative analysis of the data was carried out using the Student’s t-test (two paired and unpaired). The significance levels were p* ≤0.05 (5%); p** ≤0.01 (1%); p*** ≤0.001 (0.1%). Data were presented as means of the number (n) of indicated experiments.

## Results

### Quality Control Experiment

To test the efficiency of the new method, a CD31-positive mouse endothelial cell line (H5V) and a CD31-negative mouse colon cancer cell line (CT26) were mixed at a ratio of 1∶20. After a simulated digestion with collagenase (Figure 1B, second bar) and magnetic bead separation (Figure 1B, third bar) according to the method described in Figure 1A, the percentage of CD31 positively stained H5V cells remained unchanged compared to that of untreated H5V cells. The mean surface density of CD31 - which remained high after collagenase digestion (Figure 1C, second bar) - dropped to 17% following CD31-biotin-streptavidin competition (Figure 1C, third bar). Despite the low density of free CD31 molecules on the cell surface of H5V cells after CD31-biotin-streptavidin competition, the recovery rate of H5V cells was more than 70% after the complete separation procedure (Figure 1D). It is assumed that the recovery rate of primary ECs are higher because the mean cell surface density of CD31 on heart, repair blastema and tumor ECs (85±8, 479±14, 211±67) are higher compared to that of H5V cells (31±4). Flow cytometric analysis revealed that no contaminating CD31-negative tumor (CT26) cells were present in the CD31-positive fraction (data not shown). These findings demonstrate that it is possible to recover CD31-positive ECs from a mixture of different cells with high efficiency and purity.

### Determination of the Yield of ECs from Different Tissues in Mice of Different Ages

**Figure 2:**
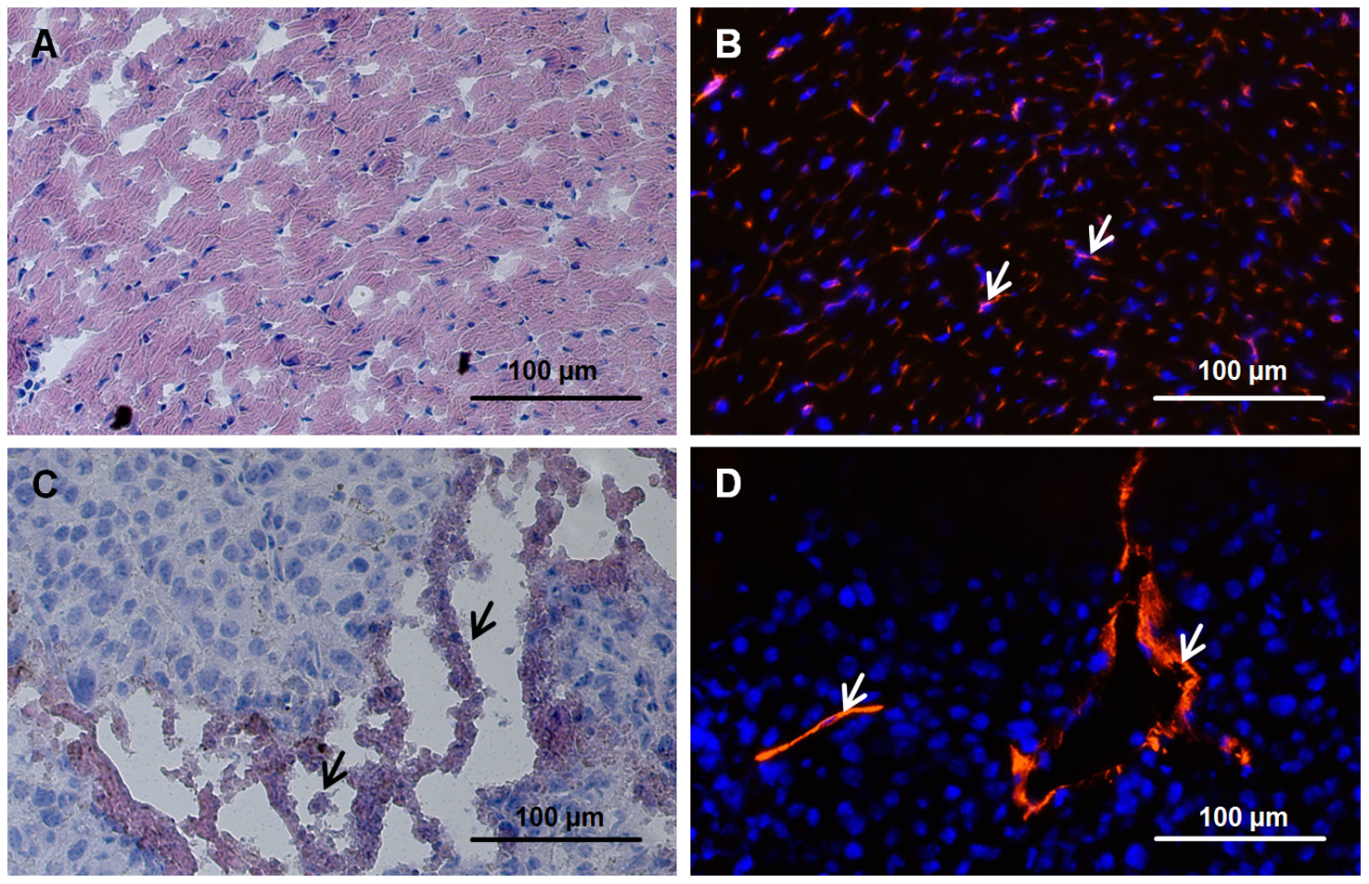
Localization of necrosis/apoptosis-fraction and capillaries in heart and tumor (B16-F0, size between 0.3–0.4 cm^3^). (A, C) H&E staining of heart and tumor, respectively. Arrows indicate the fraction of necrotic/apoptotic areas. (**B, D**) CD31 immunostaining of heart and tumor, respectively. Arrows indicate CD31-positive ECs (red).

**Figure 3:**
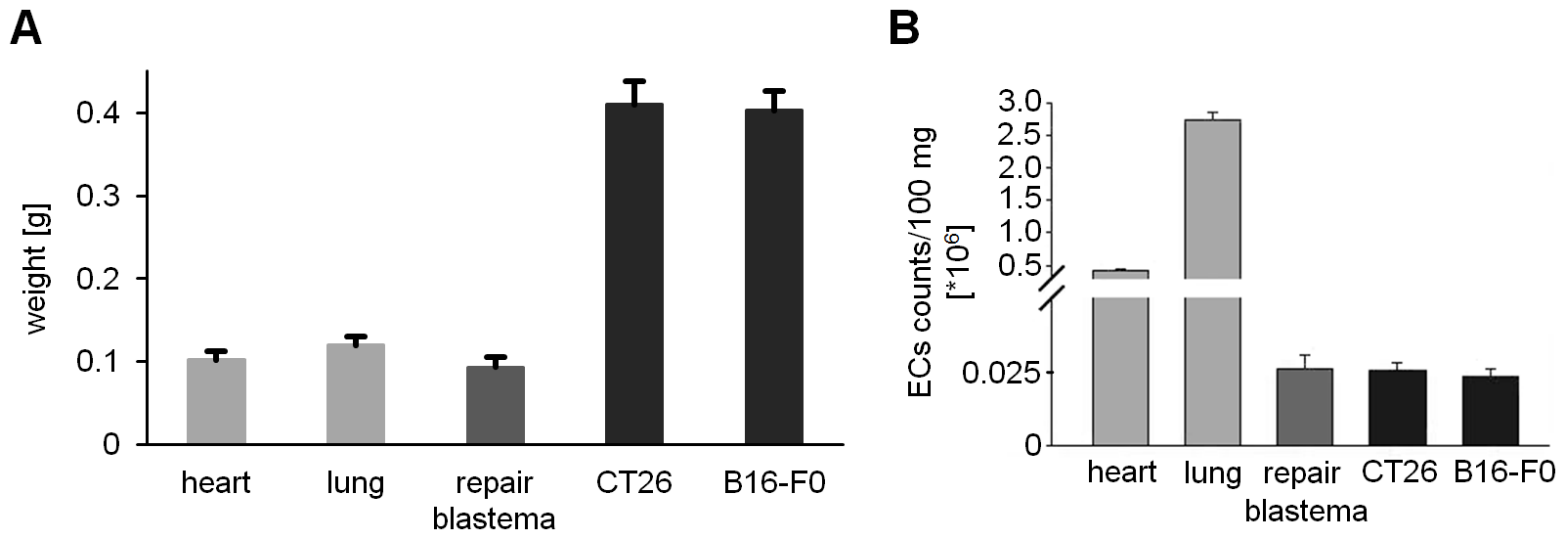
Weight of various tissues and counts of isolated ECs. (**A**) Summary of the total weight of separate tissues; weight of heart (n = 10) and lung (n = 3) taken from 13–15 week-old female mice; weight of repair blastemas (n = 10) taken from a balloon which was induced by using Freund’s adjuvant/PBS-emulsion; weight of CT26 (n = 10) and B16-F0 (n = 10) tumors which were taken when the size was ranging between 0.3 and 0.4 cm^3^. (**B**) Corresponding isolated EC counts were calculated per 100 mg tissue.

To determine the distribution and density of capillaries and the fraction of necrotic and apoptotic areas, sections of hearts and tumors of mice were analyzed by CD31 immunofluorescence and H&E staining. Viable tumor tissue is characterized by compact tissue with high cell densities, whereas necrosis is characterized by increased eosin staining and a bulky extracellular matrix. In sections of the heart, capillaries and myocardial tissues were homogeneously distributed. CD31-positive vessels were found in close proximity to cardiomyocytes (Figure 2A, B). In contrast, the capillary densities in tumor sections were lower and vessels were predominantly found in the viable outer borders, but not in the necrotic center of the tumor (Figure 2C, D). In line with these findings, the yield of ECs derived from heart and lung tissues was much higher compared to that of tumors and repair blastemas (Figure 3A, B).

An important feature of the newly established method is that comparable amounts of viable, primary microvascular ECs that became adherent to plastic could be isolated from tumor and normal tissues of young (5 weeks old) and old mice (up to 100 weeks old) (Table 1). Repeated isolation procedures revealed that it was possible to isolate approximately 0.45×10^6^ CD31-positive ECs from one heart, approximately 3×10^6^ CD31-positive ECs from one lung, approximately 0.025×10^6^ CD31-positive ECs from one repair blastema and approximately 0.1×10^6^ CD31-positive ECs from one tumor (Figure 3A, B).

**Table 1:** Number of viable heart ECs, heart weight and EC adherence after two days as a function of mice age (n = 2).

age of mouse [weeks]	sex	weight of heart [g]	ECs counts directlyafter isolation	total adherent ECsafter 2 days
5	f	0.07	350,000	60,000
20	f	0.11	400,000	40,000
50	f	0.12	490,000	40,000
100	m	0.14	550,000	50,000

### ECs Derived from Heart, Lung, Repair Blastema and Tumor Differ in Size

**Figure 4:**
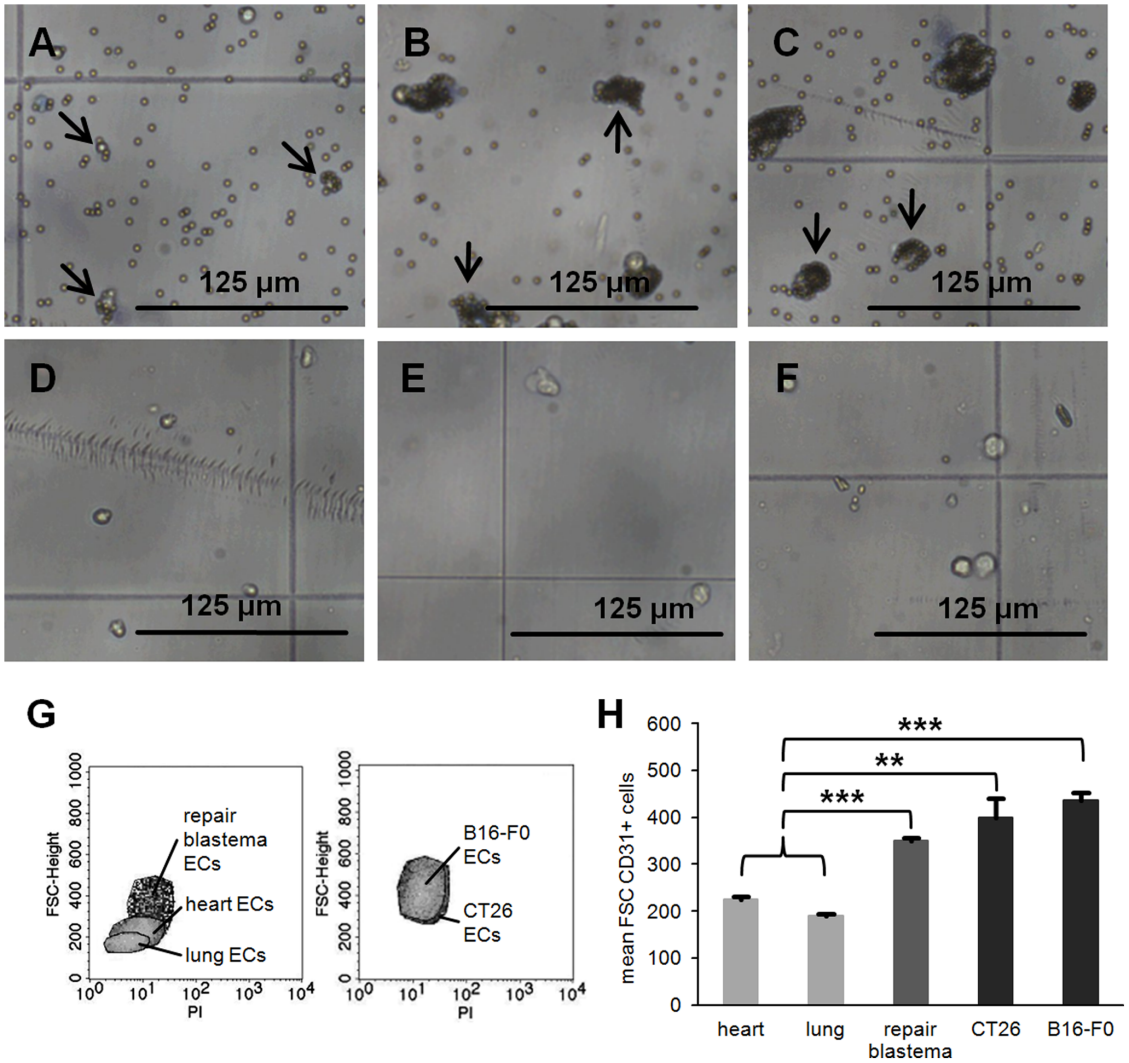
Size of primary ECs isolated from different tissues. (**A–C**) Isolated ECs from heart, repair blastema and B16-F0 with bound Dynabeads. Arrows indicate specific binding of corresponding CD31-positive ECs to Dynabeads. (**D–F**) Isolated ECs from heart, repair blastema and B16-F0 after biotin-streptavidin competition without any bound Dynabeads. (**G, H**) Mean of the forward scatter (FSC) from CD31+ cells isolated from heart (n = 4), lung (n = 3), repair blastema (n = 2), CT26 (n = 4) and B16-F0 (n = 4) measured using flow cytometry. Asteriks represent significantly different values (p** ≤0.01; p*** ≤0.001).

After digestion and magnetic bead separation of CD31-positive and CD31-negative cells, primary ECs were inspected microscopically. In most cases, ECs derived from the heart and lung bound 2–5 micro-beads (Figure 4A), whereas ECs derived from repair blastema and tumors (CT26, B16-F0) bound more than 20 beads (Figure 4B, C). After biotin-streptavidin competition most ECs were completely separated from the magnetic beads (Figure 4D–F). The size of ECs from repair blastema and both tumors were twice the size of ECs derived from normal tissues (heart, lung). The diameter of heart and lung ECs ranged between 1.5 and 2 μm, whereas that of repair blastema and tumors ECs ranged from 3 to 4 μm. Determination of the height of the forward scatter (FSC-height) by flow cytometry (Figure 4G, H) confirmed that the mean FSC-height of ECs from repair blastema (350±7) and tumors of different entities (CT26∶400±40, B16-F0∶437±17) were about twice the size of ECs derived from heart (225±6) and lung (189±6) tissues. The purity of ECs derived from heart, lung and tumors was almost 100%.

### Typical EC Cell Surface Markers Differ on Primary ECs of Different Origins

**Figure 5:**
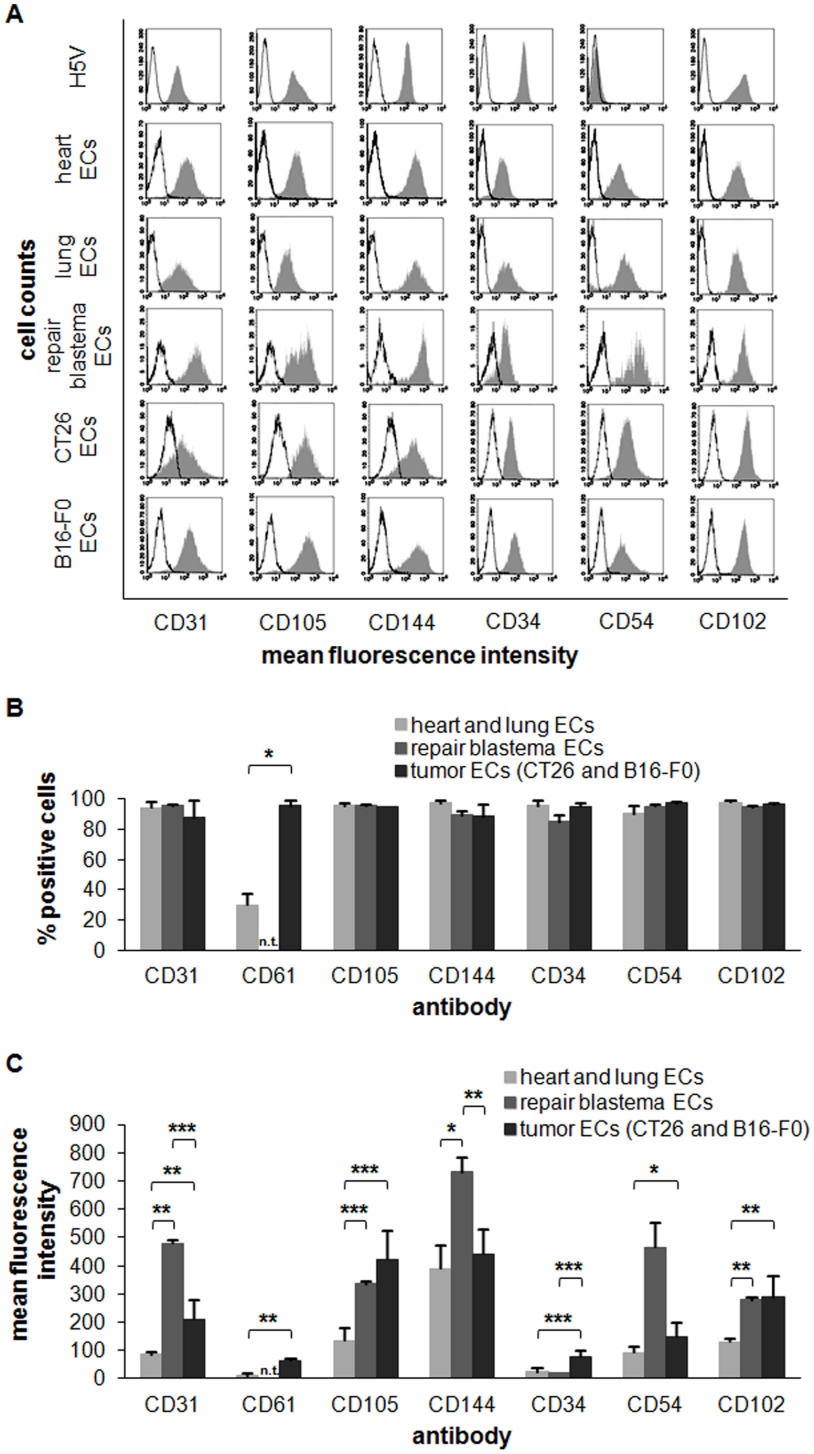
Identification and characterization of ECs derived from different origin. (**A**) Identification of control EC line H5V and primary ECs isolated from heart, lung, repair blastema, CT26 and B16-F0 using flow cytometry with EC-specific antibodies directed against CD31, CD105, CD144, CD34, CD54 and CD102. (**B, C**) Proportion of positively stained cells and mean fluorescence intensity values (mfi) (CD31, CD61, CD105, CD144, CD34, CD54, CD102) from normal ECs (heart (n = 5), lung (n = 3)), repair blastema ECs (n = 2) and tumor ECs (CT26 (n = 3), B16-F0 (n = 3)). Asteriks represent significantly different values (p* ≤0.05; p** ≤0.01; p*** ≤0.001).

Primary ECs directly after isolation from heart, lung, repair blastema and tumor were characterized by flow cytometric analysis using EC-specific antibodies directed against CD31, CD105, CD144, CD34, CD54 and CD102. The leukocyte marker CD45, which was used as control, was not detectable on the population of separated ECs (data not shown). A representative histogram of each antibody staining pattern is depicted in Figure 5A, a summary of the proportion of positively stained cells in Figure 5B and the mean fluorescence intensity values in Figure 5C are illustrated. The proportion of cells stained positively for CD31, CD105, CD144, CD34, CD54 and CD102 is nearly 100% (94±4) independent of the origin of the tissue from which the ECs were generated. In contrast, the proportion of the proliferation marker CD61 was high on tumor ECs (96±4) and low on heart ECs (30±7). For the analysis of the ECs derived from the repair blastema, all CD31-negative cells were removed using a special gating-strategy. For this, only CD31-positive cells were visualized in the histograms. In general, the cell surface density of typical EC markers is higher in ECs derived from tumors than that of normal (heart, lung) tissues, except for CD144. The marker densities of CD31 (p = 0.006), CD105 (p = 0.0004), CD34 (p = 0.0005), CD54 (p = 0.03) and CD102 (p = 0.001) were all significantly increased (two to four-fold) in tumors as compared to ECs derived from normal tissue (Figure 5C). The expression density of CD31 (p = 0.007) on ECs derived from repair blastema was six-fold higher and that of CD105 (p<0.001) twice as high as that found on normal (heart, lung) tissues. The expression density of CD34 on repair blastema ECs was similar to that found on heart or lung ECs, whereas the expression density of CD102 was more similar to that seen in tumor ECs. Furthermore, ECs from the repair blastema show a two-fold higher expression of CD144 (p = 0.01) and a five-fold higher expression density of CD54 (p = 0.1) compared to that of ECs from normal tissues (heart and lung).

Comparative flow cytometric analysis revealed that - except for the case of CD144 (360±83 vs 244±6) - the density (mfi) of typical cell surface markers of ECs such as CD31 (86±6 vs 87±2), CD105 (153±11 vs 155±17), CD34 (22±1 vs 22±1), CD54 (73±20 vs 84±11), CD102 (128±14 vs 137±18) were expressed at similar densities on ECs of hearts of young (5 weeks) and old (85 weeks) mice (Table 2). This suggests that expression patterns are organ specific and remain stable on ECs of young and old mice. The density of the EC marker CD144 which enables tight junctions is substantially higher in ECs of young mice compared to that of old mice. This might be related to the fact that the microvasculature becomes leaky in aged mice.

**Table 2:** Mean fluorescence intensity (mfi) of CD31, CD105, CD144, CD34, CD54 and CD102 of heart ECs in dependency of the age of mice (n = 2).

age of mice [weeks]	mfi CD31	mfi CD105	mfi CD144	mfi CD34	mfi CD54	mfi CD102
5	86±6	153±11	360±83	22±1	73±20	128±14
85	87±2	155±17	244±6	22±1	84±11	137±18

**Figure 6:**
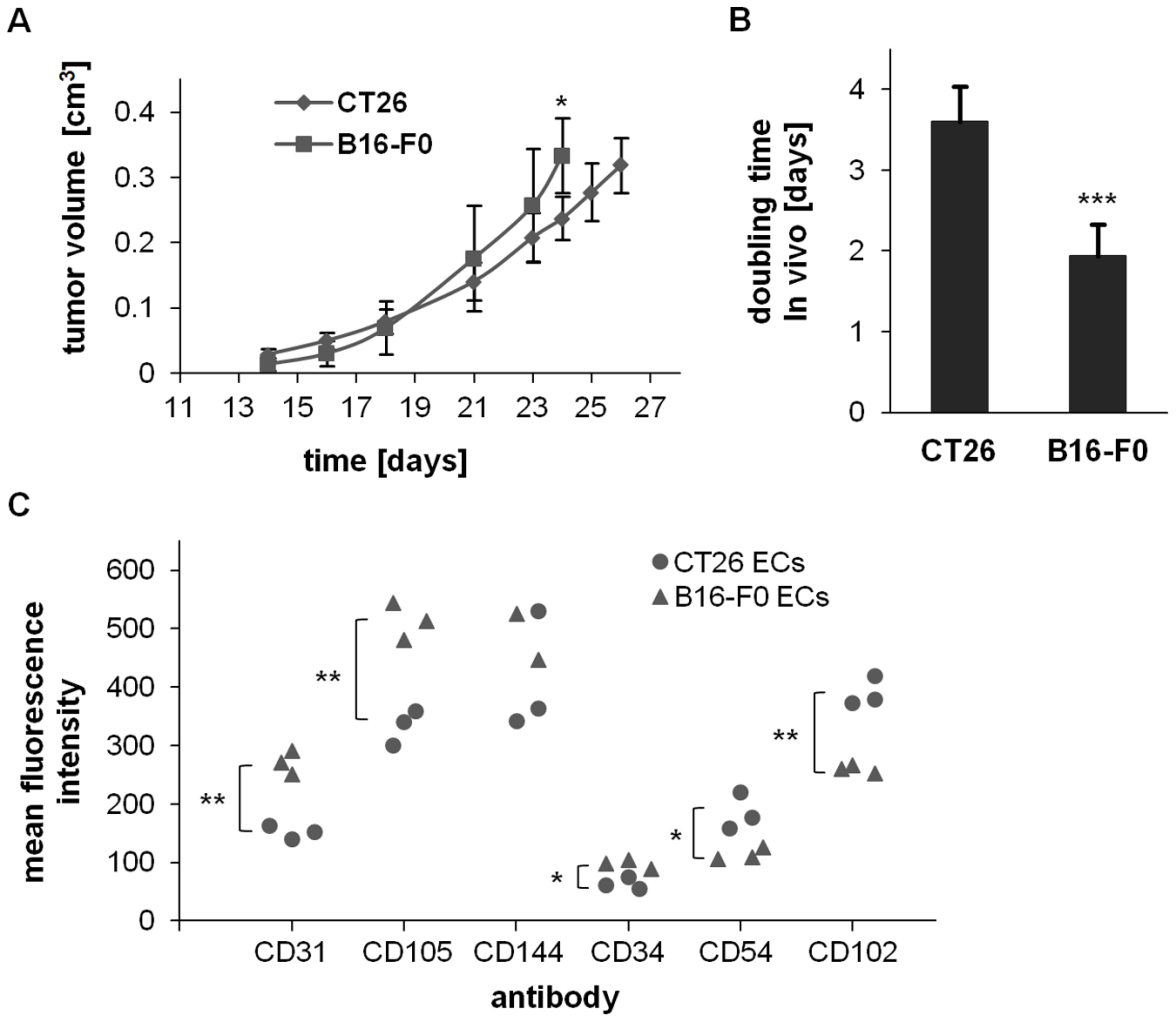
Differences in expression of EC markers derived from slow- and fast-growing tumors (CT26 and B16-F0). (**A, B**) Growth curves and doubling times of CT26 (n = 15) and B16-F0 (n = 15), *in vivo*. Asteriks represent significantly different values (p* ≤0.05; p*** ≤0.001). (**C**) Mean fluorescence intensity values (CD31, CD105, CD144, CD34, CD54, CD102) from slow-growing tumor ECs (CT26 (n = 3)) and fast-growing tumor ECs (B16-F0 (n = 3)). Asteriks represent significantly different values (p* ≤0.05; p** ≤0.01).

The B16-F0 tumors grow faster compared to CT26 tumors *in vivo* (Figure 6A). The mean doubling times for CT26 and B16-F0 tumors was 3.6 and 1.9 days (p<0.001), respectively (Figure 6B). The expression densities of the EC markers CD31 (p = 0.002), CD105 (p = 0.002) and CD34 (p = 0.01) were higher on ECs derived from fast-growing B16-F0 tumors, whereas the expression densities of the cellular adhesion molecule CD54 (p<0.05) and CD102 (p = 0.008) were found to be elevated in the slow-growing CT26 tumor (Figure 6C).

**Figure 7:**
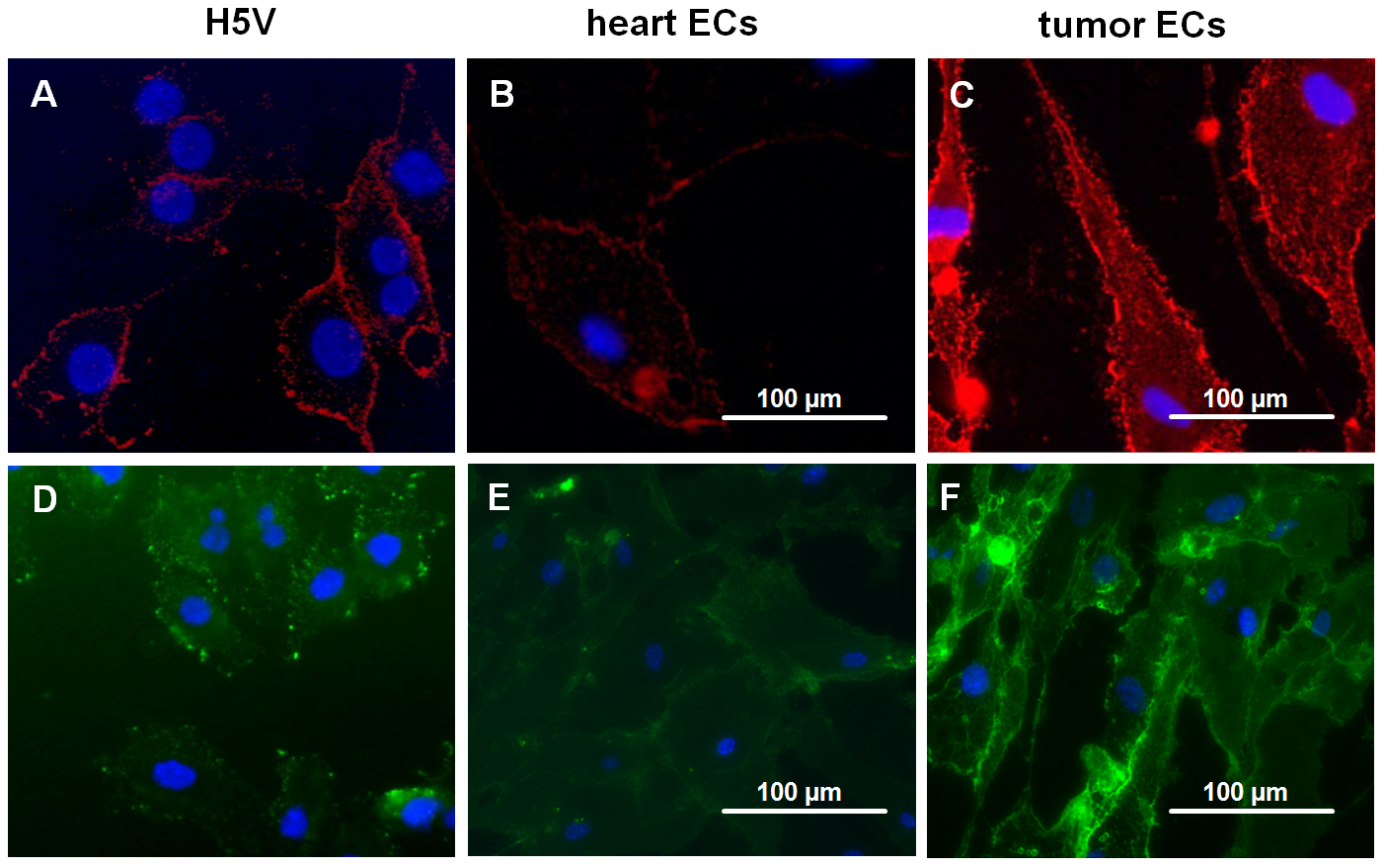
Identification of ECs by immunostaining. (**A–C**) CD31 staining of H5V, primary heart and tumor ECs (B16-F0). (**D–F**) Isolectin B4-staining of H5V, primary heart and tumor ECs (B16-F0).

Comparative *in vitro* immunofluorescence studies showed that the fluorescence intensity of CD31 and isolectin B4 on heart ECs (Figure 7B, E) was much weaker compared to that on tumor ECs (Figure 7C, F). As a control, both markers were also used to stain the murine endothelial cell line H5V (Figure 7A, D) since this reflects more exactly the staining pattern of the tumor than the normal primary ECs.

### The Morphology and Migration of Normal and Tumor Derived ECs Differ *in vitro*

**Figure 8:**
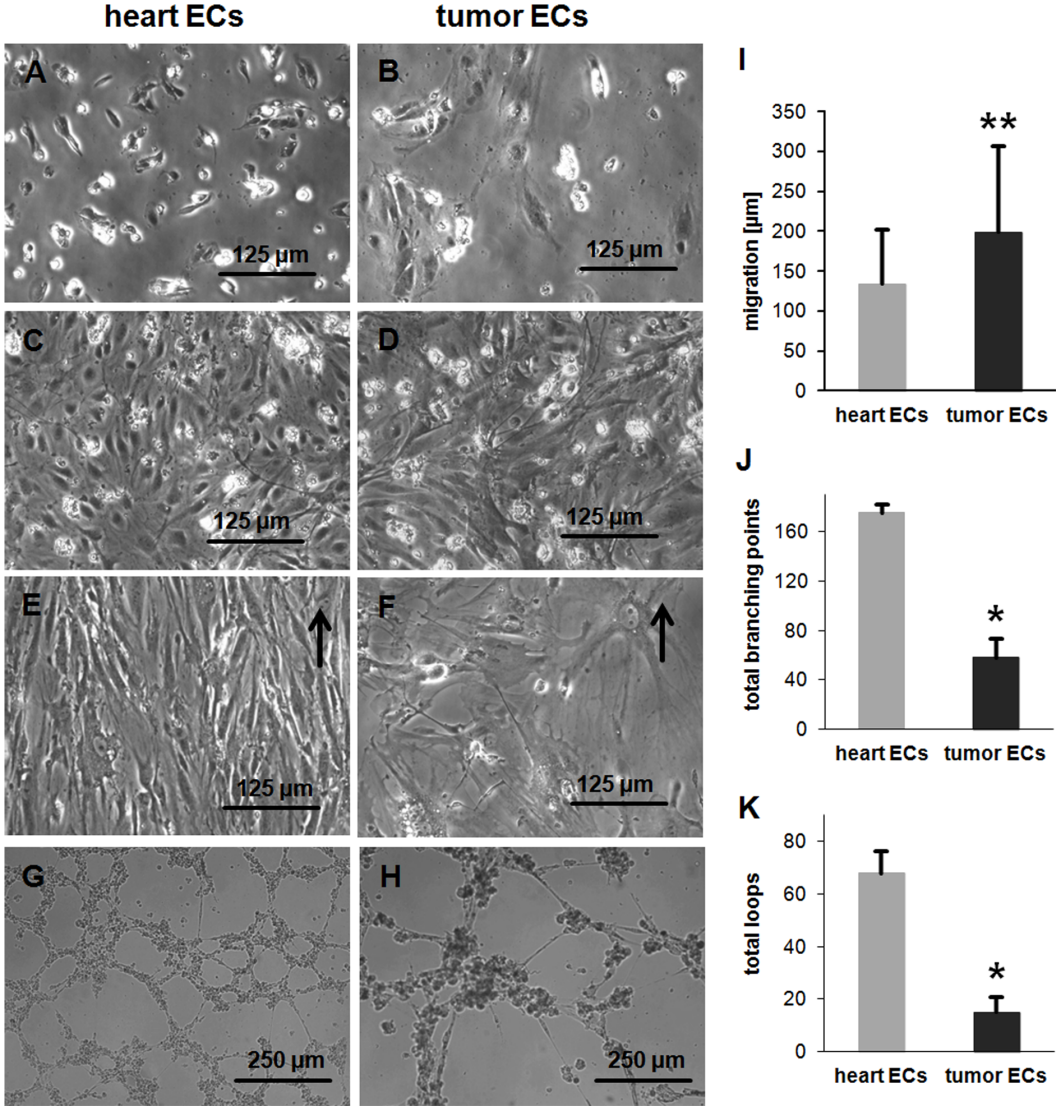
Phenotypic and functional characteristics of primary heart and tumor ECs (B16-F0), *in vitro*. (A, B) Adherent heart and tumor ECs after 2 days. (**C, D**) Adherent heart and tumor ECs after 7 days. (**E, F**) Heart and tumor ECs under flow conditions (4 ml/min) after 4 days. Before starting the flow cells were incubated for 2 days under static conditions and 1 day under low flow conditions (2 ml/min). Arrows indicate flow direction. (**G, H**) Vascular tube formation of heart and tumor ECs on matrigel. (**I**) Migratory capacity of heart and tumor ECs. Asteriks represent significantly different values (p** ≤0.01). (**J, K**) Number of total branching points and total loops of heart and tumor ECs on matrigel. Asteriks represent significantly different values (p* ≤0.05).

Primary ECs derived from normal (heart) and tumor (B16-F0) tissues of mice became adherent 1–2 days after seeding (Figure 8A, B) and the diameter of ECs increased over the following 5 days until the cells reached 100% confluence (Figure 8C, D). In contrast to ECs derived from normal tissues, adherent tumor ECs were found to be larger, to spread faster and to distribute in a more chaotic pattern compared to those of normal tissues (heart). The migratory capacity of heart ECs (134±68 μm) is significantly lower compared to that of tumor ECs (199±108 μm) (Figure 8I). Although heart and tumor ECs became confluent cell divisions were, as expected, not detectable (video S2, S3), in contrast to the endothelial cell line H5V (video S1).

### Normal But not Tumor Derived ECs Align Under Flow Conditions

The cultivation of primary heart and tumor ECs under permanent shear stress allows the physiological conditions in blood vessels to be simulated. Therefore, 2 days after allowing the cells to attach, ECs were cultured under constant flow conditions using the IBIDI system. To avoid detachment of cells the flow was started at a rate of 2 ml/min for the first day, after which it was increased to 4 ml/min. After two days, heart ECs started to elongate in the direction of the flow. Four days later most heart ECs showed an alignment in the direction of the flow (Figure 8E). In contrast, ECs derived from tumors did not align in the direction of the flow (Figure 8F).

### Primary ECs Derived from Normal and Tumor Tissues Differ in Their Capacity to form Tubes

Due to the larger size of tumor ECs compared to normal tissue ECs, a higher number of heart (80,000 cells/well) than tumor (20,000 cells/well) ECs was seeded into the matrigel. The numbers used mirror the minimum number of cells that are required to allow a tube formation within a defined area (Figure 8G, H). Tubes formed by tumor ECs show a significantly smaller number of branching points and loops (58±16, 15±6) per image compared to that of ECs derived from heart tissues (176±6, 68±8) (Figure 8J, K).

## Discussion

Previously characterizations of primary ECs *in vitro* were based on ECs isolated from young mice within the age range of a few days up to two weeks. ECs derived from older mice (>2 weeks) did not become adherent, could not be maintained or expanded in cell culture, and underwent apoptotic cell death within a few days. The standard method for isolation of ECs is the use of magnetic beads that are coupled with a specific antibody directed against EC cell markers. However, beads bound to isolated ECs represent a steric disruption, and thus prevent attachment of freshly isolated primary ECs to plastic surfaces, thus disturbing subsequent experiments.

Up to now, ECs could only be isolated from growing tissues of very young mice that have the capacity to proliferate and therefore, have the chance to get rid of the attached beads with increasing cell divisions. A comparative analysis of cell surface markers by flow cytometry is only possible after the beads have been detached from the ECs. The results of analysis using these *in vitro* cultured ECs may not be representative of the functional status of normal ECs *in vivo,* directly after their isolation. Even HUVECs and transformed endothelial cell lines such as H5V provide limited results, since their doubling time *in vitro* does not reflect the very low proliferation rate of ECs in healthy tissues of adult mice [3]. In contrast, the proliferation rate of ECs under pathological conditions such as wound healing, inflammation and tumors is high. It has therefore been assumed that proteins expressed in ECs of resting and growing tissues may be different. By using the technique described in this paper, primary ECs could be isolated from young and old mice in the absence of attached micro-beads in order to characterize them in the stationary phase without many cell divisions since isolation, *in vitro*. In the present study, heart and lung were used as examples of stable, non-proliferating normal tissues, the repair blastema as a rapidly proliferating normal tissue, and CT26 and B16-F0 tumors as proliferating malignant tissues. Isolated cells could be clearly identified as ECs from each individual tissue due to the expression of a variety of different typical EC surface markers such as CD31, CD105, CD144, CD34, CD54 and CD102. Many studies have used only one marker for the characterization of ECs. This is however, not sufficient to characterize ECs because each individual marker alone can also be expressed by other cell types. The platelet endothelial cell adhesion molecule 1 (PECAM-1)/CD31 for example is also expressed by platelets, monocytes, macrophages and neutrophils in addition to ECs [11]–[13]. Endoglin/CD105 is expressed on vascular ECs, hematopoietic cells and on several different normal and tumor cell types [14]. Vascular endothelial (VE)-cadherin/CD144 is a specific adhesion molecule of ECs and the major component of adherent junctions [15], [16]. Sialomucin/CD34 is mainly expressed by hematopoietic progenitor cells and by ECs from blood vessels, but not by ECs from lymphatic vessels [17]–[19]. ICAM-1/CD54 is expressed constitutively at low levels on vascular ECs and also on leukocytes [20]. ICAM-2/CD102 is expressed constitutively on lymphocytes, monocytes, platelets and ECs. These data indicate that the identification of ECs requires the staining of the cells with more than just one antibody. All antibodies depicting markers which are typical for ECs were expressed on nearly 100% of the isolated ECs, irrespectively of the tissue from which they were derived, whereas CD61, a proliferation marker was expressed highly only on ECs that were derived from proliferating tissues.

The expression density of angiogenesis-related markers such as CD31, CD105, CD144, and CD102 is higher in freshly isolated ECs of proliferating tissues (tumors, repair blastema) as compared to non-proliferating normal tissues such as heart and lung. Even so, the expression of the stemness-related marker CD34 is elevated in tumor-derived ECs, but not in that of repair blastemas, whereas the adhesion molecule CD54 was found to be elevated in ECs of benign and malignant proliferating tissues but not in non-proliferating normal tissues. The highest density of CD31 and CD144 was found in ECs isolated from the repair blastema, which may account for the very rapid angiogenesis exhibited by this tissue.

Primary ECs isolated from CD31 deficient mice showed a decreased cell migration and a reduced capillary morphogenesis [21]. Blocking of CD31 inhibited the growth and vascularization of tumors in mice and inhibited the EC tube formation, *in vitro* [22]. However, CD31 knockout mice developed a normal functional vascular system during embryonic development, but a decreased angiogenesis in inflammation [23]. CD61 was upregulated in tumor vasculature and it was shown that depletion of CD61 transiently inhibited tumor growth and angiogenesis in mice [24], [25]. CD105 expression is increased in ECs during wound healing, developing embryos, inflammatory tissues and solid tumors [26], [27]. All of these processes are involved in angiogenesis and are associated with the receptor of the transforming growth factor-β complex (TGF-β). A high expression of CD105 on tumor ECs correlates with a reduced survival of patients [28], [29]. ECs from CD144-deficient mice form primitive vascular structures, which are unstable during further development and result in embryonic death [30]–[32]. Furthermore, antibodies against CD144 reduced angiogenesis and tumor growth *in vivo* [33]–[35]. The present study shows that high expression densities of CD31, CD61, CD105 and CD144 on freshly isolated ECs correlate with increased angiogenic capacity. Additionally, CD105 has been described as being capable of preventing apoptosis in hypoxic ECs and leading to an enhanced survival of ECs under hypoxic stress [36]. This may be the reason that CD105 is expressed at higher levels on tumor ECs compared to ECs isolated from repair blastema.

The expression density of CD34 in mouse embryonic blood vessels is high in pre-endothelial cells and in vessels formed by vasculogenesis and angiogenesis, but low in vessels, which are involved in the process of coalescence to form larger vessels [37]. In the early phase, CD34 knockout mice with transplanted B16-F1 melanoma cells showed a decreased, and then later an increased tumor growth caused by a reduced capacity of CD34−/− hematopoietic cells [38]. In this present study the fact that CD34 is expressed at a higher level on tumor ECs could be due to circulating endothelial progenitor cells, which might be involved in the vasculogenesis of the tumor.

CD54 is also up-regulated by inflammatory cytokines in various cell types and may increase leukocyte extravasation in inflammation [20], [39]. CD54 inhibition in mouse melanomas using CD54 antisense oligonucleotides resulted in fewer metastases in the lungs [40]. Antibodies against CD54 also blocked the invasion of metastatic human breast cancer cells *in vitro* [41]. In contrast to CD54, the expression of CD102 is not increased by inflammatory cytokines [42]. Experiments in CD102-deficient mice have shown that CD102 on ECs is required for angiogenesis and is involved in survival and cell migration, but not in EC proliferation [43]. The present study shows that the expression of CD102 is increased on ECs from growing tumors and repair blastemas, whereas the expression of CD54 is increased mainly in physiological wound healing.

The higher expression of CD31, CD105 and CD34 on ECs from the B16-F0 tumor correlates with the faster tumor growth compared to that of CT26 tumors. In contrast, the higher expression of CD54 and CD102 on ECs from CT26 correlates with the higher metastatic behavior of CT26 compared to B16-F0 in lung, when cells were subcutaneously injected in syngeneic mice [44], [45].

Tumor ECs are larger, have a significantly higher migratory capacity and distribute in a more chaotic pattern in cell culture compared to ECs derived from normal tissue, *in vitro*. Primary ECs from non-proliferating and proliferating tissue differ in their functional properties, *in vitro*. Tube formation assays showed that tumor ECs have a smaller number of branching points and loops compared to that of normal ECs and, in contrast to normal tissue ECs, tumor-derived ECs show no tendency to align under flow conditions.

In summary, the novel method presented here, provides a powerful tool for the purification of viable primary microvascular ECs of different origins which can be used to analyze organ-specific immunological interactions and functional changes in the interplay of existing and evolving ECs from benign and malignant tissues.

Our results provide evidence indicating that freshly isolated primary ECs from different tissues differ in many respects. These differences may be related to the different functions of the microvasculature of the tissues from which the ECs have been isolated. The expression density of the different markers on ECs might have an impact on the proliferation rate of ECs, the permeability for metastasis or the involvement of circulating endothelial progenitor cells.

## Supporting Information

Video S1 **Movie illustrating the migratory and proliferative capacity of the heart EC cell line H5V for 33 h (30 frames per second).**
(MPG)

Video S2 **Movie illustrating the migratory capacity of primary heart ECs for 33 h (30 frames per second).**
(MPG)

Video S3 **Movie illustrating the migratory capacity of primary tumor ECs for 33 h (30 frames per second).**
(MPG)
